# Endurance Exercise Ability in the Horse: A Trait with Complex Polygenic Determinism

**DOI:** 10.3389/fgene.2017.00089

**Published:** 2017-06-28

**Authors:** Anne Ricard, Céline Robert, Christine Blouin, Fanny Baste, Gwendoline Torquet, Caroline Morgenthaler, Julie Rivière, Nuria Mach, Xavier Mata, Laurent Schibler, Eric Barrey

**Affiliations:** ^1^Institut National de la Recherche Agronomique, AgroParisTech, Université Paris Saclay, Département Sciences du Vivant, UMR 1313 Génétique Animale et Biologie IntégrativeJouy-en-Josas, France; ^2^Institut Français du Cheval et de l'Equitation, Département Recherche et InnovationExmes, France; ^3^Ecole Nationale Vétérinaire d'AlfortMaisons Alfort, France

**Keywords:** genotyping, exercise, endurance, horse, SORCS3, SLC39A12, KCNQ1OT1, GWAS

## Abstract

Endurance horses are able to run at more than 20 km/h for 160 km (in bouts of 30–40 km). This level of performance is based on intense aerobic metabolism, effective body heat dissipation and the ability to endure painful exercise. The known heritabilities of endurance performance and exercise-related physiological traits in Arabian horses suggest that adaptation to extreme endurance exercise is influenced by genetic factors. The objective of the present genome-wide association study (GWAS) was to identify single nucleotide polymorphisms (SNPs) related to endurance racing performance in 597 Arabian horses. The performance traits studied were the total race distance, average race speed and finishing status (qualified, eliminated or retired). We used three mixed models that included a fixed allele or genotype effect and a random, polygenic effect. Quantile-quantile plots were acceptable, and the regression coefficients for actual vs. expected log_10_ *p*-values ranged from 0.865 to 1.055. The GWAS revealed five significant quantitative trait loci (QTL) corresponding to 6 SNPs on chromosomes 6, 1, 7, 16, and 29 (two SNPs) with corrected *p*-values from 1.7 × 10^−6^ to 1.8 × 10^−5^. Annotation of these 5 QTL revealed two genes: sortilin-related VPS10-domain-containing receptor 3 (*SORCS3*) on chromosome 1 is involved in protein trafficking, and solute carrier family 39 member 12 (*SLC39A12*) on chromosome 29 is active in zinc transport and cell homeostasis. These two coding genes could be involved in neuronal tissues (CNS). The other QTL on chromosomes 6, 7, and 16 may be involved in the regulation of the gene expression through non-coding RNAs, CpG islands and transcription factor binding sites. On chromosome 6, a new candidate equine long non-coding RNA (*KCNQ1OT1* ortholog: opposite antisense transcript 1 of potassium voltage-gated channel subfamily Q member 1 gene) was predicted *in silico* and validated by RT-qPCR in primary cultures of equine myoblasts and fibroblasts. This lncRNA could be one element of the cardiac rhythm regulation. Our GWAS revealed that equine performance during endurance races is a complex polygenic trait, and is partially governed by at least 5 QTL: two coding genes involved in neuronal tissues and three other loci with many regulatory functions such as slowing down heart rate.

## Introduction

A large body of epidemiological evidence suggests that regular, moderate, aerobic exercise is positively correlated with good health (Neilson et al., 2009; Bishwajit et al., 2016; Kanagasabai et al., 2017). However, the physiological and cellular mechanisms that underlie this correlation have not been extensively characterized. The identification of genetic variants associated with the ability to perform long bouts of aerobic exercise could be one means of tackling this question. The horse is an interesting physiological model in this context because different breeds are specialized in all types of exercise. For instance, Arabian horses are well adapted to endurance racing, and are able to run at an average speed of 20 km/h or more for up to 160 km (in bouts of 30–40 km). This level of performance is based on intense aerobic metabolism, adaptation of the cardiorespiratory system, effective body heat dissipation, and maintenance of homeostasis.

In humans, the cardiorespiratory system's adaptation to training is influenced by genetic factors and significant heritability. It has been reported that nine intragenic single nucleotide polymorphisms (SNPs) in three genes (*YWHAQ, RBPMS*, and *CREB1*) are linked to adaption of the heart rate response to steady-state, sub-maximal exercise at 50 watts (Rankinen et al., 2012). The three genes are involved in genomic regulation. Improvement of submaximal aerobic capacity with training (as measured by changes in oxygen consumption and power output) is also partly associated with 14 SNPs in two candidate genes on chromosome 13 (mitochondrial intermediate peptidase, encoded by *MIPEP*, and sarcoglycan gamma, encoded by *SGCG*) (Rice et al., 2012). In humans, the five following genes (reviewed by Pérusse et al., 2013) are thought to partly explain endurance exercise ability and the response to training: acetyl-coenzyme A synthase long-chain family member 1 (*ACSL1*), ATPase aminophospholipid transporter (*ATP8A2*), GS homeobox protein 1 gene (*GSX1*), uncoupling protein 1 and 3 (*UCP2* and *UCP3*).

In contrast, only a few equine genes associated with exercise ability have been identified (Barrey, 2010; Petersen et al., 2013). In thoroughbreds (a horse bred for racing over distances of 1,200–2,600 m), various SNPs in the myostatin gene (*MSTN*) are significantly associated with galloping speed over short, medium and long distances (Hill et al., 2010). In the French Trotter (bred for harness/trotting races over 1,600–4,100 m), SNPs in the *DMRT3* gene are associated with the neurosensorial coordination ability required for fast trotting (Andersson et al., 2012). The first genotype is linked to fast trotting in young horses, the second is linked to fast trotting in older horses, and the third is linked to poor trotting ability (Ricard, 2015). Although few genes related to exercise ability are known, heritability estimates of endurance race performance indicate the presence of a significant genetic component (*h*^2^ = 0.28 for average race speed and *h*^2^ = 0.06 for finishing position) (Ricard and Touvais, 2007). Taken as a whole, the results of genetic studies of human endurance exercise and the significant heritability observed in equine endurance competitions suggest that genetic variants are associated with the specific physiological adaptations required for equine endurance exercise (i.e., the ability to canter at least 20 km/h for 8 h). This is likely to be especially true for pure-bred Arabians (the most successful breed in international endurance competitions).

Hence, the objective of the present genome-wide association study (GWAS) was to identify genetic determinants (i.e., SNPs) of the ability to perform endurance exercise in Arabian horses competing in high-level events. Briefly, we identified five quantitative trait loci (QTL) associated with the performance traits after detecting six significant SNPs on chromosomes 1, 6, 7, 16, and 29. Two of the five QTL are coding genes involved in neuronal tissues and three QTL are non-coding sequences with many putative regulatory functions such as cardiac recovery control.

## Methods

### Horse population, blood samples, and ethical aspects

The blood samples used in the present study were collected during national-level French endurance races (distance: 90–160 km) in 2011 and 2012. Additional DNA samples were obtained from a sample collection owned by a parentage testing laboratory. After quality tests, 597 individual samples were genotyped. Their phenotypes including age, breed, gender and performance traits are described in Table S1. The great majority of these horses (72%) were Arabians and crossed Arabians through the sire (89%). 85% of the horses were born between 1998 and 2004, 3% were younger (born in 2005) and 12% were older (born between 1990 and 1997). The gender ratio was well balanced (49% females).

The genetic structure of the studied horse population can be described by the following data: the 597 horses were the progeny of 285 sires and 542 mares which make an average family size of 2.1 progeny/sire and 1.1 progeny/mare. The sires were progeny of 195 grand-fathers (3.1 sires/grand-father) and 15 of them produced 10 sires. The mares were issued from 187 grand-fathers (2.9 mares/grand-father) and 13 of them produced between 10 and 55 mares.

The study was approved by the Animal Use and Care Committee at Alfort Veterinary School and the University of Paris-Est (ComEth Anses/ENVA/UPEC; approval number 12/07/11-1. The consent obtained from all the horse owners was informed, written and signed by each owner prior to any study procedures.

### Genotyping

All horses were genotyped using the equine SNP-74K chip (Illumina, San Diego, CA, USA). After quality control (MAF ≥ 1%; call rate ≥ 80%; *p*-value test Hardy-Weinberg >10^−8^), 56,200 SNPs were selected from autosomal chromosomes.

### Trait: endurance racing performance

Performances in French endurance races from 2002 to 2011 (38,473 results and 7,363 horses) were assessed with regard to three performance traits: speed (standardized by race), total distance covered, finishing status (qualified, eliminated or retired). These performances were corrected for fixed environmental effects: gender (female, male, gelding), age (6–12 years and over 12 years), race (2,263 races, no race effect for the distance trait) and averaged by taking into account their heritability and repeatability presented in Table 1 (Ricard and Touvais, 2007). This yielded a unique pseudo-performance value for each trait computed according to the statistical method described in Tables S1, S2. The pseudo-performance value was weighted by the number of observations per horse and by genetic parameters. The weighting factor was referred to as the equivalent number of performances (ENP) (Table S3).

**Table 1:** Genetic parameters used in breeding evaluations of endurance horses for speed (S), distance covered (D), and finishing status (F).

**Criteria**	**Speed**	**Distance**	**Finishing status**
Speed	**0.20** *0.39*	0.52	0.64
Distance	0.54	**0.10** *0.20*	0.85
Finishing status	0.51	0.91	**0.10** *0.20*

*Heritabilities in the diagonal (bold), the genetic correlations above the diagonal (underlined) and repeatabilities (italic) in the diagonal, and the phenotypic correlations between the traits below the diagonal*.

## Statistical models

Three complementary models were applied, in order to maximize the chance of QTL detection at three levels (SNPs, genotypes and haplotypes).

### Models 1 and 2: mixed models for detecting SNP alleles and genotypes

These were mixed models with a single allele SNP effect:

(1) and (2) $$\begin{array}{r} y=1\mu +\text{x}\beta +\text{Zu}+\text{e} \end{array}$$

The vector y is the vector for the racing pseudo-performance traits in the 597 genotyped horses. The μ is the mean of racing pseudo-performance. In model (1) **x** is the vector of genotypes of the SNP analyzed (i.e., 0, 1 or 2 according to the copies number of the reference allele) and “beta” is the allele effect of the SNP. In that case, the allele effect is additive. In model (2), **x** is an incidence vector (i.e., 0 or 1) and “beta” is the vector of the effect of the 3 genotypes for the SNP. In that case, dominance effects are authorized.

The *u* is the vector for a random polygenic effect with $V\left(u\right)=A\sigma_{u}^{2}$, where A is the relationship matrix between genotyped horses, (calculated from 9,481 horses with ancestors), *e* is the vector for residuals with, for racing performance, $V\left(e\right)=D\sigma_{e}^{2}$, and *D* is a diagonal matrix with diagonal coefficients $d_{kk}=\frac{1}{m_{k}}$ for each genotyped horse *k* with *m*_*k*_ as the ENP. The polygenic variance was $\sigma_{u}^{2}=h^{2}\sigma_{y}^{2}$. For racing traits, $\sigma_{e}^{2}=\left(1-r\right)\sigma_{y}^{2}$, where r is the repeatability of the performance trait. Heritability and repeatability were estimated from the full set of 7,363 horses and the full model (including gender, age and race effects) (Table 1). A Student's test of the null hypothesis (β = 0) against the alternative (β ≠ 0) was performed for each SNP for the model 1. Estimates were obtained using BLUPF90 software (Misztal et al., 2002).

### Model 3 for haplotype detection

Model 3 used phased data. Haplotypes were obtained using PHASEBOOK (Druet and Georges, 2010). PHASEBOOK is a software package for obtaining phased haplotypes in a population with a high number of related animals. First, haplotypes are reconstructed from pedigree information (Mendelian segregation rules and linkage information) using LinkPHASE. The gaps are then filled by applying a hidden Markov model from BEAGLE (Browning and Browning, 2007). These programs were run using the parameters recommended in Druet and Georges (2010). First, LinkPHASE was run once after setting the probability threshold of parental origin to 1. Secondly, DAGPHASE was run to attribute randomly missing alleles. Thirdly, BEAGLE was iteratively used with DAGPHASE to construct an optimal directed acrylic graph (DAG). DAGPHASE was then used to sample haplotypes from these DAGs and improve the latter. BEAGLE was then run again, and so on. BEAGLE was run with a scale of 2.0, a shift of 0.1 and five iterations. The BEAGLE outputs correspond to the haplotypes and the hidden states used to construct the haplotypes. We used a mixed model with two random effects:

(3) $$\begin{array}{r} y=1\mu +\text{W}\eta +\text{Zu}+\text{e} \end{array}$$

where y, u, and e were the same as in model 1, W is an incidence matrix which links a horse to its pair of hidden haplotypes states, so that the sum of each row is 2, and **η** is the vector for haplotypes at the SNP location. Haplotypes were defined from the three closest SNPs upstream of the reference SNP and the three closest SNPs downstream (i.e., with 7 SNPs in total). The number of haplotypes varies from one SNP to another. The **η** effect was considered to be random, and its variance was estimated using REMLF90 (Misztal et al., 2002). The statistical test used was the likelihood-ratio test, which compares the likelihoods obtained with and without a haplotype effect. The test's distribution is not known but has previously been shown to be close to one half of a 0-degree of freedom (df) χ^2^ distribution plus one-half of a 1-df χ^2^ distribution for a single position (Self and Liang, 1987). The *p*-values were computed using this distribution.

### Significance threshold

Significance threshold for *p*-values was calculated for each test after checking the QQ plots. The threshold for the type 1 error was set to 5% after Bonferroni correction. The number of independent tests used for Bonferroni correction was calculated according to Goddard (2009) and Goddard et al. (2011), in order to obtain the equivalent number of independent markers *M*_*e*_ as a function of the LD:

$$\begin{array}{l} \frac{1}{56200*56200}\overset{56200}{\underset{i=1}{\sum }}\overset{56200}{\underset{j=1}{\sum }}r_{ij}^{2}=\frac{1}{M_{e}} \end{array}$$

where $r_{ij}^{2}$ is the LD between SNPs *i* and *j* (i.e., a correlation between genotypes). Lastly, the threshold was set to 5%/*M*_*e*_.

### QTL annotations: data mining around the SNPs

#### Candidate genes

The location of each SNP was compared with the EquCab 2.0 reference genome (available in Ensembl database). For example, we found the SNP BIEC2_11782 in the gene *SORCS3*. (http://www.ensembl.org/Equus_caballus/Gene/Summary?db=core;g=ENSECAG00000008241; r = 1:25204207-25768630;*t* = ENSECAT00000008738).

#### CNV regions

We used the CNV data from a meta-analysis of the horse genome (Ghosh et al., 2014).

#### Gene enrichment analysis

Close microRNA (miRNA) and other gene loci located 4 Mbp upstream or 4 Mbp downstream of the significant SNPs were automatically identified using the EquCab 2.0 reference genome (Wade et al., 2009) and miRBase (Kozomara and Griffiths-Jones, 2014). The list of genes was submitted to DAVID software (Huang et al., 2009) in order to assess putative gene function enrichment. The list of miRNAs located close to the SNPs was drawn up, and their predicted gene targets were identified by using TARGET SCAN and MIRDB (Wang and El Naqa, 2008). The corresponding regulated pathways were identified by using DIANA Tools (Vlachos et al., 2012).

#### Long non-coding RNA

The DNA sequence located 4 Mbp upstream and 4 Mbp downstream of the significantly associated intergenic SNPs was systematically aligned against all the long non-coding RNA (lncRNA) present in the lncRNAdb database (http://www.lncrnadb.org/) by using BLASTN (Quek et al., 2014). Thus, it was possible to detect partial similarities with a prediction score; it is known that lncRNAs are poorly conserved between species but that their regulatory functions (and perhaps their secondary structures) are better conserved.

#### CpG islands

The DNA sequence located 0.5 Mbp upstream and 0.5 Mbp downstream of the significant intergenic SNPs was screened to detect CpG islands, using the EMBOSS Cpgplot algorithm (Rice et al., 2000).

#### Tanscription factors binding sites

The DNA sequence located 1 Mbp upstream and 1 Mbp downstream of the significant intergenic SNPs was systematically compared with the positions of all transcription factors (TF) binding sites (531401) and potential TF ligands predicted by TRANSFAC Pro method for the EquCab2 reference genome (http://www.gene-regulation.com/pub/databases.html).

### Use of RT-qPCR to detect equine lncRNA orthologs of *KCNQ1OT1*

In order to validate the *in silico* prediction of a novel equine lncRNA ortholog of *KCNQ1OT1*, we used a specific tool (CLC Workbench, CLC Bio, MA, USA) to design three pairs of primers at various points in the aligned antisense sequence (2,364 nt) (Table S4). The 18S rRNA was used as an endogenous reference gene because it was expressed to the same extent in all tested samples. Total RNA was extracted from primary cultures of equine myoblasts (*n* = 3) and fibroblasts (*n* = 3). Reverse transcription was undertaken using an efficient reverse transcriptase (Superscript VILO cDNA Synthesis Kit, ThermoFisher Scientific), according to the manufacturer's instructions. Real-time quantitative PCR was carried out in a 7,500 machine (Applied Biosystems) using the Syber Green method (Power Syber Green kit, Applied Biosystems). The average relative expression of the myoblast vs. fibroblast was calculated by the formula:

Fold change = 2^−ΔΔCt^

where ΔΔCt = (myoblast sample − Ct 18S) − (mean Ct fibroblast − Ct 18S).

## Results

### Overview

Three quantitative criteria (traits) were used to measure equine performance in endurance races: average race speed, total ride distance and finishing status (i.e., qualified or eliminated). By using three complimentary polygenic models, our GWAS revealed five significant quantitative trait loci (QTL) corresponding to 6 SNPs on chromosomes 6, 1, 7, 16, and 29 (two synonymous SNPs on Chr 29) with corrected *p*-values from 1.7 × 10^−6^ to 1.8 × 10^−5^ (Table 2). We name the 5 QTL according to their chromosome position i.e., QTL#6, 1, 7, 16, and 29 in the following part of manuscript. The large distribution of the hits suggested that the endurance performance trait is highly polygenic. The most significant hit was the QTL#6 associated to total distance trait and with a *p*-value = 1.5 × 10^−6^ and a percentage of variance explained of 1.27 % (Table 2).

**Table 2:** Significant SNPs associated to performance traits in endurance ride, ranked according to their significance (Corrected *p*-values).

**QTL#^a^**	**SNP identification**	**Chr:Position^b^**	**Traits^c^**	***p*-value**	**Model^d^**	**Alleles, genotypes, haplotypes**	**Frequency (%)**	**Effect^e^**	**Variance explained^f^(%)**
6	BIEC2_1022884	6: 79312770	Distance	1.5 × 10^−6^	1	A/G	15 – 85	0.22 / 0	1.27
			Finish status	2.3 × 10^−6^	1	A/G	15 – 85	0.22 / 0	1.24
			Distance	9.2 × 10^−6^	2	AA/AG/GG	72 – 26 – 2	0.22 / 0 / −0.22	1.20
			Finish status	1.4 × 10^−5^	2	AA/AG/GG	72 – 26 – 2	0.22 / 0 / −0.22	1.09
1	BIEC2_11782	1: 25715334	Speed	1.7 × 10^−6^	3	12 haplotypes	NA	See note^g^	1.10
16	BIEC2_363958	16: 79466436	Finish status	1.4 × 10^−5^	2	CC/AC/AA	6 – 30 – 64	−0.24 / 0 / −0.23	1.19
			Distance	2.0 × 10^−5^	2	CC/AC/AA	6 – 30 – 64	−0.25 / 0 / −0.22	1.29
7	BIEC2_977605	7: 6283266	Finish status	1.8 × 10^−5^	1	G/A	75 – 25	0.16 / 0	0.97
29	BIEC2_755603	29: 17580597	Finish status	1.8 × 10^−5^	2	AA/AC/CC	11 – 39 – 50	−0.05 / 0 / −0.22	0.43
	BIEC2_755604	29:17580992	Finish status	1.8 × 10^−5^	2	GG/GA/AA	11 – 39 – 50	−0.05 / 0 / −0.22	0.43

a *Quantitative trait loci (QTL) number: this number was used to identified the QTL in the manuscript and tables. It was defined according to the corresponding chromosome location*.

b *Chromosome (Chr) and nucleotide position*.

c *The performance traits were the Distance of the ride, Status at finish (qualified or eliminated) and averaged Speed during the ride*.

d *Models: 1 = mixed model with SNP allele effect and polygenic effect; 2 = mixed model with SNP genotype effect and polygenic effect; 3 = mixed model with random 5 SNPs haplotype effects and polygenic effect*.

e *Effect on phenotype of alleles or genotypes expressed in unit phenotypic standard deviation*.

f *Variance explained in percentage of phenotypic variance*.

g *See Supplementary Material Figure S1*.

For all SNPs, our systematic data mining included gene annotations, genomic, non-coding and epigenetic prediction methods. The five QTL were linked to these genomic, non-coding RNA or epigenetic elements (Table 3). Three QTL have intronic positions in two well-conserved genes: sortilin-related VPS10-domain-containing receptor 3 (*SORCS3*) on chromosome 1 (QTL#1), and solute carrier family 39 member 12 (*SLC39A12*, coding for a protein also known as ZIP-12) on chromosome 29 (QTL#29). The three other QTL are intergenic and thus, we sought to assess regulatory functions by screening for microRNAs (miRNAs), miRNA targets, long non-coding RNAs (lncRNAs), transcription factor (TF) binding sites and CpG islands. Further details about the annotations of each QTL are presented below.

**Table 3:** Summary of the annotations of SNPs significantly associated with endurance performance traits.

**QTL#^a^**	**SNP identification**	**Gene loci identified by the SNP**	**Transcription factor (TF) binding sites (BS) within 1 Mbp**	**Number of CpG islands within 0.5 Mbp**
6	BIEC2_1022884	candidate lncRNA^b^ *KCNQ1OT1*, miRNA eca-miR-763	5 BSs, 20 TFs (NFκb)	6 within 20–34 kb
1	BIEC2_11782	*SORCS3* intron#16^c^ miRNA eca-miR-146b	1 BS, 23 TFs	5 within 50 kb
7	BIEC2_977605		1 BS, 20 TFs	2 within 11 kb
16	BIEC2_363958	CNV^d^miRNA eca-miR-1289	11 BSs, 34 TFs (PPPARγ, c-Myc, NF1)	
29	BIEC2_755603	*SLC39A12* intron#51^e^	17 BSs, 134 TFs (PPARγ, NF1, MyoD)	
	BIEC2_755604			

a *Quantitative trait loci (QTL) number: this number was used to identified the QTL in the manuscript and tables. It was defined according to the corresponding chromosome location of the SNPs*.

b *KCNQ1OT1 lncRNA: long non-coding RNA opposite strand transcript 1 for the KCNQ1 gene*.

c *SORCS3: Sortilin-related VPS10-domain-containing receptor 3*.

d *CNV: Copy number variation*.

e *SLC39A12: Solute carrier family 39 member 12*.

### GWAS quality control

**Figure 1:**
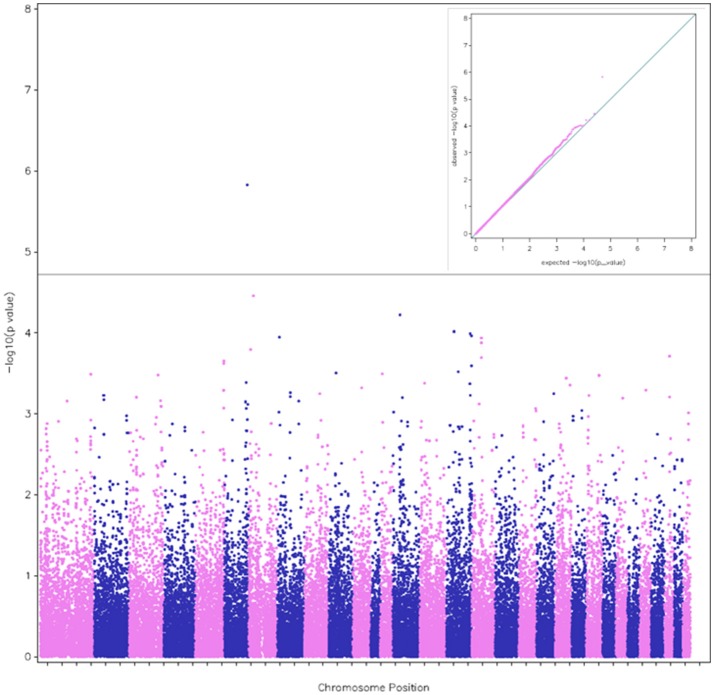
A Manhattan plot of the genome-wide associations with race distance. The plot was calculated from model (1), which detected the SNP BIEC2_1022884 on Chr 6. The red line indicates the Bonferroni-corrected significance level (*p* = 1.9 × 10^−5^). The alternative colors blue and pink indicate the successive chromosome (Chr) positions from Chr 1 to 31 (autosomal chromosomes). The corresponding quantile-quantile (QQ) plot of observed vs. expected −log10(p) values is shown in the inset.

**Figure 2:**
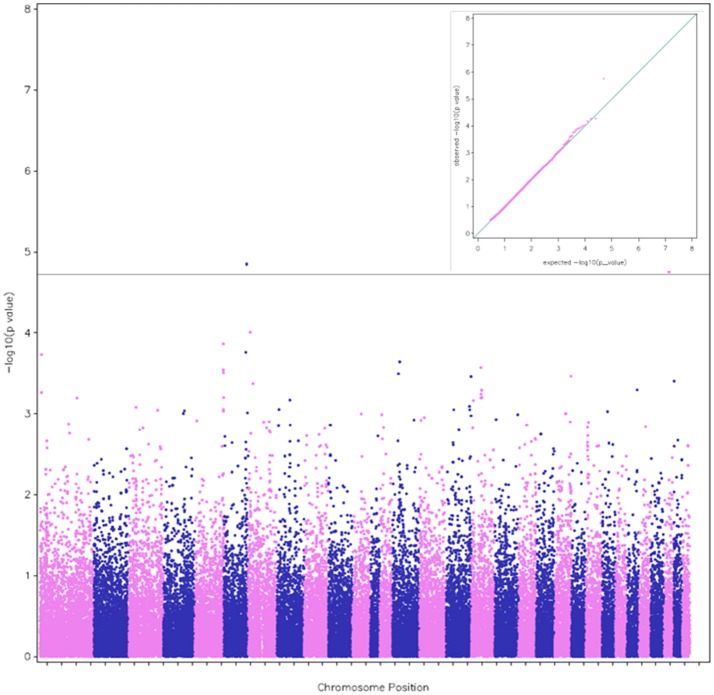
A Manhattan plot of the genome-wide associations with finishing status (qualified or eliminated) in endurance races. The plot was calculated from model (2), which detected the SNP BIEC2_1022884 on Chr 6, the SNP BIEC2_363958 on Chr 16 and the two nearby SNPs BIEC2_755603 and BIEC2_755604 on Chr 29. The alternative colors blue and pink indicate the successive chromosome positions from Chr 1 to 31. The corresponding QQ plot of observed vs. expected −log10(p) values is shown in the inset.

**Figure 3:**
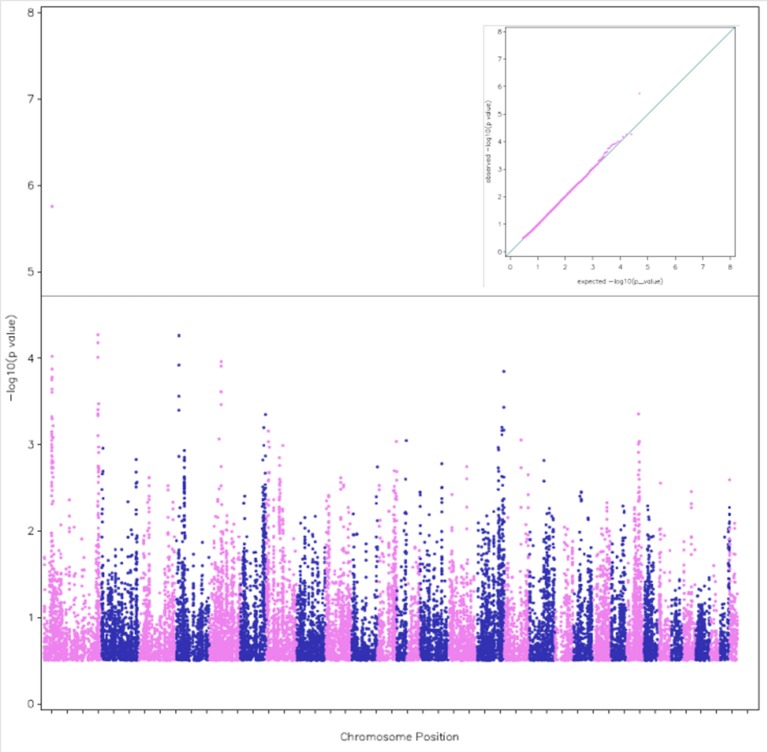
A Manhattan plot of the genome-wide associations with average race speed. The plot was calculated from model 3 which detected the SNP BIEC2_11782 on Chr 1. The alternative colors blue and pink indicate the successive chromosome positions from Chr 1 to 31. The corresponding QQ plot of observed vs. expected −log10(p) values is shown in the inset.

We drew up quantile-quantile (QQ) plots as a guide to the validity of the obtained *p*-values and the presence or absence of an underlying population structure that might not have been taken into account in our models. All the QQ plots gave acceptable results for the distribution of the test statistics: the regression coefficients for actual vs. expected log_10_ *p*-values ranged from 0.865 to 1.055 for the performance traits, indicating that the population structure was correctly taken into account by the three mixed models with polygenic effect (Figures 1–**3**).

### Linkage disequilibrium (LD) and the effective number of loci

**Figure 4:**
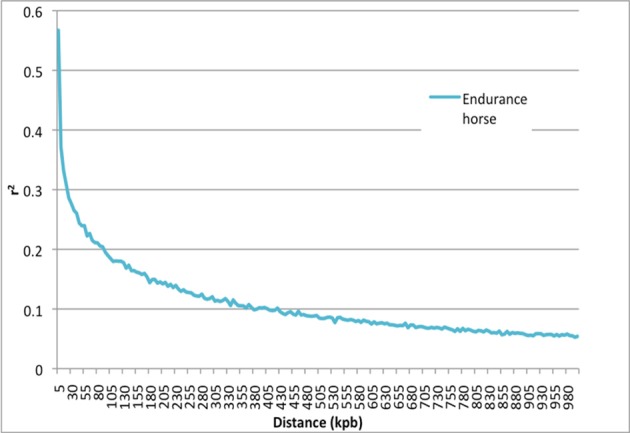
Linkage disequilibrium (*r*^2^) calculated in endurance horses population (*n* = 597). The horses were Arabians and crossed Arabians. Each point corresponds to the mean for all pairs of SNPs over 5 kbp.

Figure 4 shows the LD, as measured by the mean *r*^2^ for syntenic SNP pairs against a map distance of up to 1 Mbp. All available pairs of SNP were used and grouped by 5 kb increments. In order to be able to compare our results with those obtained in other breeds, we only analyzed SNPs with a minor allele *frequency* (MAF) > 5% (*n* = 50,311). The mean *r*^2^ at the mean distance (39.8 kb) between adjacent SNPs was 0.260. The mean of *r*^2^over all available pairs per chromosome was 0.009937 and (assuming the absence of LD between chromosomes) 0.0003897 over all available pairs. Hence, the effective number of loci was 2,566, which gave a significant threshold of *p* < 1.9 × 10^−5^.

### Detection of SNPs associated with endurance performance traits

According to the threshold chosen for the Bonferroni-corrected *p*-value, the GWAS revealed 5 QTL (#6, 1, 7, 16, and 29 according to their chromosome positions) with 6 significant SNPs (Table 2). Two close SNPs were located on chromosome 29. The most significant hit was QTL#6 (Corrected *p*-values: 1.5 × 10^−6^). The QTL#6 and #16 were associated to total distance of the race (Corrected *p*-values: 1.5 to 9.2 × 10^−6^), the QTL #6, 7, 16, and 29 were associated to finishing status (Corrected *p*-values: 2.3 × 10^−6^ to 1.8 × 10^−5^), and QTL#1 was associated to average race speed (Corrected *p*-value: 1.7 × 10^−6^) (Table 2). Manhattan plots were computed for each trait, using the three complementary models (Figures 1–3). For each trait, we cross-checked the results in additional models. For each model, we cross-checked the results for the three traits. Although the *p*-value did not always reach the significance threshold for the alternative model (1, 2 or 3), it was always close to it. When comparing traits, the *p*-values for distance and finishing status were always similar. Each model had a good fit for different traits and locations. Models 1 and 2 gave similar results when the effect was additive, and this was enough to reach the significance threshold in both cases (as the model 2 is more stringent, due to a higher number of degree of freedom). The results for finishing status and distance were rather similar (Figures 1, 2). Model 3 detected an additional SNP (centered on 12 possible haplotypes on chromosome 1) associated with the average race speed (Figure 3 and Figure S1).

The most significant QTL#6 was found on chromosome 6, where a SNP (BIEC2_1022884) was significantly associated with distance and finishing status in models 1 and 2. The effect on performance was about a quarter of a phenotypic standard deviation (SD) per copy of the allele. The frequency of allele A (associated with a longer distance and a greater likelihood of qualification at the finish) was 15%. The QTL#1 was significantly associated with average race speed and the model 3 revealed 12 possible haplotypes (including a set of 7 SNPs centered on the SNP (BIEC2_11782) (Table 2). Three of these 12 haplotypes were particularly frequent and had a greater effect on average race speed (Table 2 and Figure S1).

The QTL#16 (SNP BIEC2_363958) was significantly associated with finishing status and was just below the threshold for distance (model 2). The favorable genotype was the AC heterozygote (frequency: 30%). The two homozygotes had the same phenotypic effect (Table 2: −0.22 to −0.25 phenotypic SD). The QTL#7 detected by SNP (BIEC2_977605) was associated with finishing status (model 1). The favorable G allele was most frequent (75%) but had a low effect (phenotypic SD: 0.16). The QTL#29 was detected by two close, fully linked (*r*^2^ = 1) SNPs (BIEC2_755603 and BIEC2_755604) were associated with finishing status (Model 2). A dominance effect was found; the heterozygote and the favorable homozygote had the same level of significance, and together had the same frequency (50%) as the unfavorable homozygote.

### Potential associations with known SNPs

We specifically checked for SNPs known to be involved in different traits in the horse: (i) the BIEC2-620109 SNP on chromosome 23 (initially involved in the detection of DMRT3 mutations that affect locomotion in horses (Andersson et al., 2012), (ii) the BIEC2-808466 and BIEC2-808543 SNPs on chromosome 3, (iii) the BIEC2-1105370, BIEC2-1105373, BIEC2-1105377, BIEC2-1105505 and BIEC21105840 SNPs on chromosome 9 (linked to height at the withers (Signer-Hasler et al., 2012), and (iv) the BIEC2-417210, BIEC2-417274, BIEC2-417372, BIEC2-417423 and BIEC2-417524 SNPs near the mutation in the equine myostatin gene (MSTN) (Hill et al., 2010) on chromosome 18. In the present GWAS, none of these SNPs had an effect on racing traits for endurance horses. Furthermore, two were not polymorphic in Arabian horses (BIEC2-620109, related to DMRT3, and BIEC2-808466, related to height at the withers), although the rare allele is found in crossed animals at a very low frequency (1.3 and 2.2%, respectively), and no homozygotes were detected in our dataset.

### SNP annotation and data mining

By using all available annotations in the equine reference genome (EquCab 2, version 87: http://www.ensembl.org/Equus_caballus/Info/Index?db=core;g = ENSECAG00000001249;*r* = 11:4215263-4215814), we first determined whether a given SNP was located within a gene or was close to a gene. Regions with gene copy number variations (CNVs) were compared with the SNPs' positions. If a candidate coding gene was not found at or within 4 Mbp of the SNP's position, we searched for putative non-coding RNAs involved in the regulome (either microRNAs already annotated on the equine genome or lncRNAs identified in other databases). Lastly, the sequence regions were used to predict TF binding sites and CpG islands. CpG islands are constituted by long C-G repeats at which cytosine methylation can indicate epigenetic regulation. The findings for each QTL are summarized in Table 3.

#### Candidate genes

On chromosome 1, the BIEC2_11782 SNP was located in intron #1 of the *SORCS3* gene (Figure S2). This 27-exon gene codes for a type I transmembrane receptor protein that is a member of the vacuolar protein sorting 10 family of receptors, which have pleiotropic functions in protein trafficking and intracellular/intercellular signaling in both neuronal and non-neuronal cells. The two SNPs on chromosome 29 (BIEC2_2755603 and BIEC2_755604) were also intronic, and were located in intron #51 of the *SLC39A12* gene (Figure S3). This 13-exon gene codes for the ZIP12a zinc transporter, which performs Zn^2+^ uptake and maintains cell zinc homeostasis in many species. Zn^2+^ is a cofactor in protein, nucleic acid, carbohydrate and lipid metabolism, and is also involved in the control of gene transcription, growth, development, and differentiation.

All the other SNPs were located outside gene loci. We analyzed the functions of all genes located within 4 Mbp of the significant SNPs. Interestingly, some enrichments of function were found in the list of genes for the following pathways directly involved in endurance exercise: mitochondrial metabolism, oxidative phosphorylation metabolism, metal ion and cation binding, and haematopoiesis (Table S5). We found a significant enrichment of mitochondrial genes around BIEC2_11782 on chromosome 1 and BIEC2_1022884 on chromosome 6 (Table 4). However, the long distances between the SNPs and the gene locations are solely compatible with epigenetic gene regulation; we found CpG islands on chromosome 6, for example (see below).

**Table 4:** Enrichment of mitochondrial genes around the SNPs associated with endurance performance traits.

**# QTL**	**SNP**	**Gene-SNP distance**	**Gene ID**	**Gene name**	**Gene description**
6	BIEC2_1186704	1,017,260	ENSECAG00000025143	CYP27B1	Mitochondrial 25 hydroxyvitamin D3-1alpha-hydroxylase
		1,213,794	ENSECAG00000022334	DCTN2	Dynactin 2 (p50)
		1,439,200	ENSECAG00000023525	NDUFA4L2	Similar to NADH dehydrogenase [ubiquinone] 1 alpha subcomplex subunit 4-like 2
		1,445,447	ENSECAG00000021295	SHMT2	Serine hydroxymethyltransferase 2 (mitochondrial)
		2,131,486	ENSECAG00000022627	ATP5B	mitochondrial ATP synthase, H+ transporting F1 complex beta subunit
		2,372,006	ENSECAG00000016568	CS	Citrate synthase
1	BIEC2_11782	1,689,257	ENSECAG00000010022	CYP17A1	Cytochrome P-450 17 alpha-hydroxylase/C17,20-lyase
		3,586,938	ENSECAG00000010039	LOC100060451	Similar to NADH dehydrogenase
		3,732,238	ENSECAG00000014658	SDC	Similar to stearoyl-CoA desaturase
		3,929,908	ENSECAG00000024865	P450 2C33v4	Similar to cytochrome P450 2C33v4

#### Regions with CNVs

On the basis of a meta-analysis of all the CNV regions identified to date in the horse genome (Ghosh et al., 2014), we found that the QTL on chromosome 16 was located within a CNV region (Table 3). The QTL #1 and 6 were located near to CNV regions but not within them.

#### MiRNAs

Four miRNA sequences (eca-miR-146b, 763, 7, and 1,289) were found in proximity to the QTL #6, 1, 7, and 16 (Table S6). The miRNAs' predicted gene targets allowed to identify more than 30 putative regulated pathways (*p*-values from 8.79 × 10^−12^ to 0.05). Among all the enriched pathways, we noticed interesting functions related to long exercise which might be regulated such as circadian rhythm (10 genes; *p* = 3.14 × 10^−7^), Wnt signaling (36 genes; *p* = 8.79 × 10^−12^), neutrophin signaling (27 genes; *p* = 1.19 × 10^−8^), ubiquitin mediated proteolysis (27 genes; *p* = 3.58 × 10^−6^), actin cytoskeleton (34 genes; *p* = 0.0002), glycosaminoglycan biosynthesis (31 genes; *p* = 2.38 10^−5^), protein processing endoplasmic reticulum (28 genes; *p* = 0.0003), gap junction (19 genes; *p* = 3.48 × 10^−9^) and tight junctions (20 genes; *p* < 0.05) (Table S7).

#### LncRNAs

**Figure 5:**
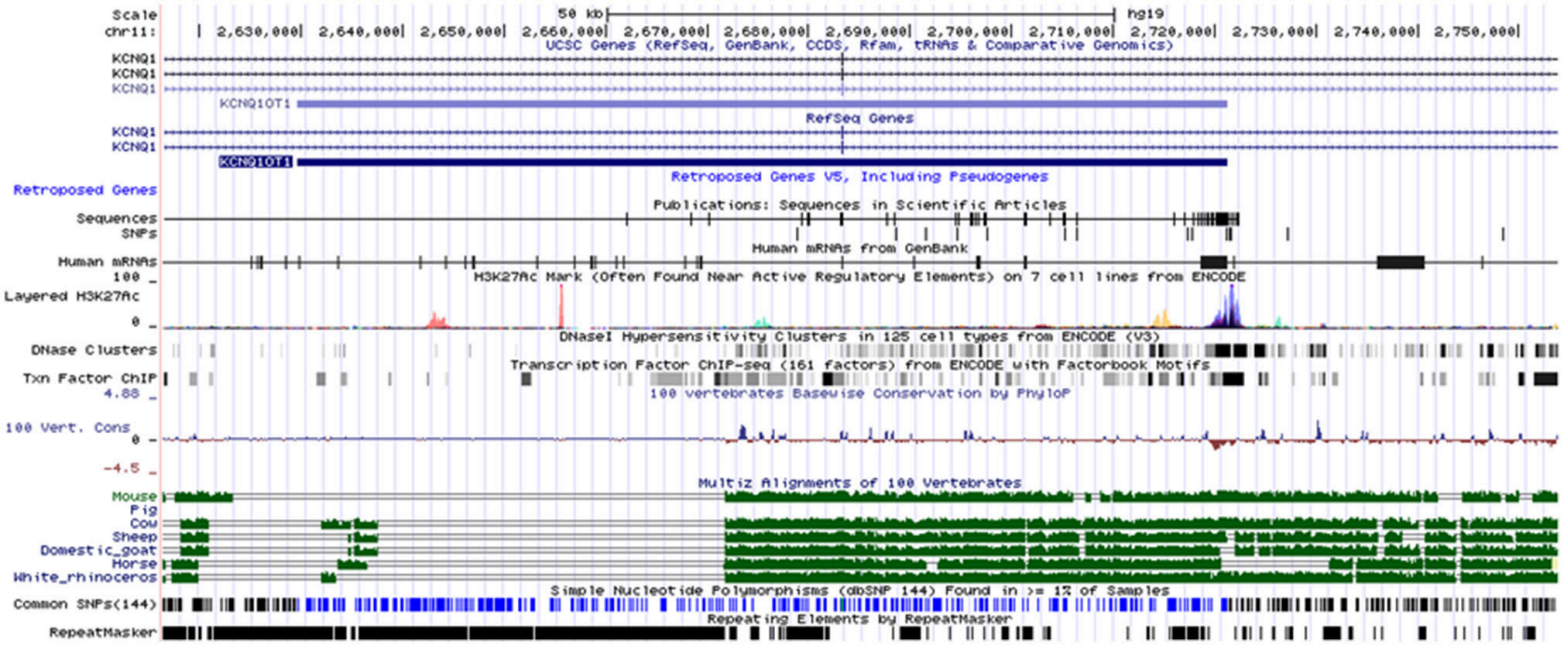
Genetic map of the human *KCNQ1OT1* lncRNA and corresponding alignments with other species. A significant alignment is observed in horse and other domesticated animals. The map is obtained from ENCODE.

For the intergenic QTL#6, 7, and 16, we performed a BLASTN search of the lncRNAdb database in order to reveal significant alignments of conserved domains. One very significant hit (score: 226; *E*-value = 10^−57^) was found on chromosome 6, where the human sequence *KCNQ1OT1* (a chromatin-interacting regulatory lncRNA) was aligned with the equine intergenic sequence [chr6: 79205561:79470270] (Figure 5). Surprisingly, this lncRNA is highly conserved among domesticated mammals and humans. In order to validate this *in silico* annotation, we used RT-qPCR (with 3′, 5′ and in-sequence pairs of primers) to detect the corresponding aligned, antisense sequence (2,364 nt; Table S4). Significant amounts of this lncRNA candidate were detected in total RNA extracted from primary cultures of equine myoblasts and fibroblasts: the *Ct* values for the three pairs of primers ranged from 27.8 to 33.6. The lncRNA candidate *KCNQ1OT1* was less strongly expressed in myoblasts than in fibroblasts, with a fold change of 0.57 (Table S8).

#### CpG islands

**Figure 6:**
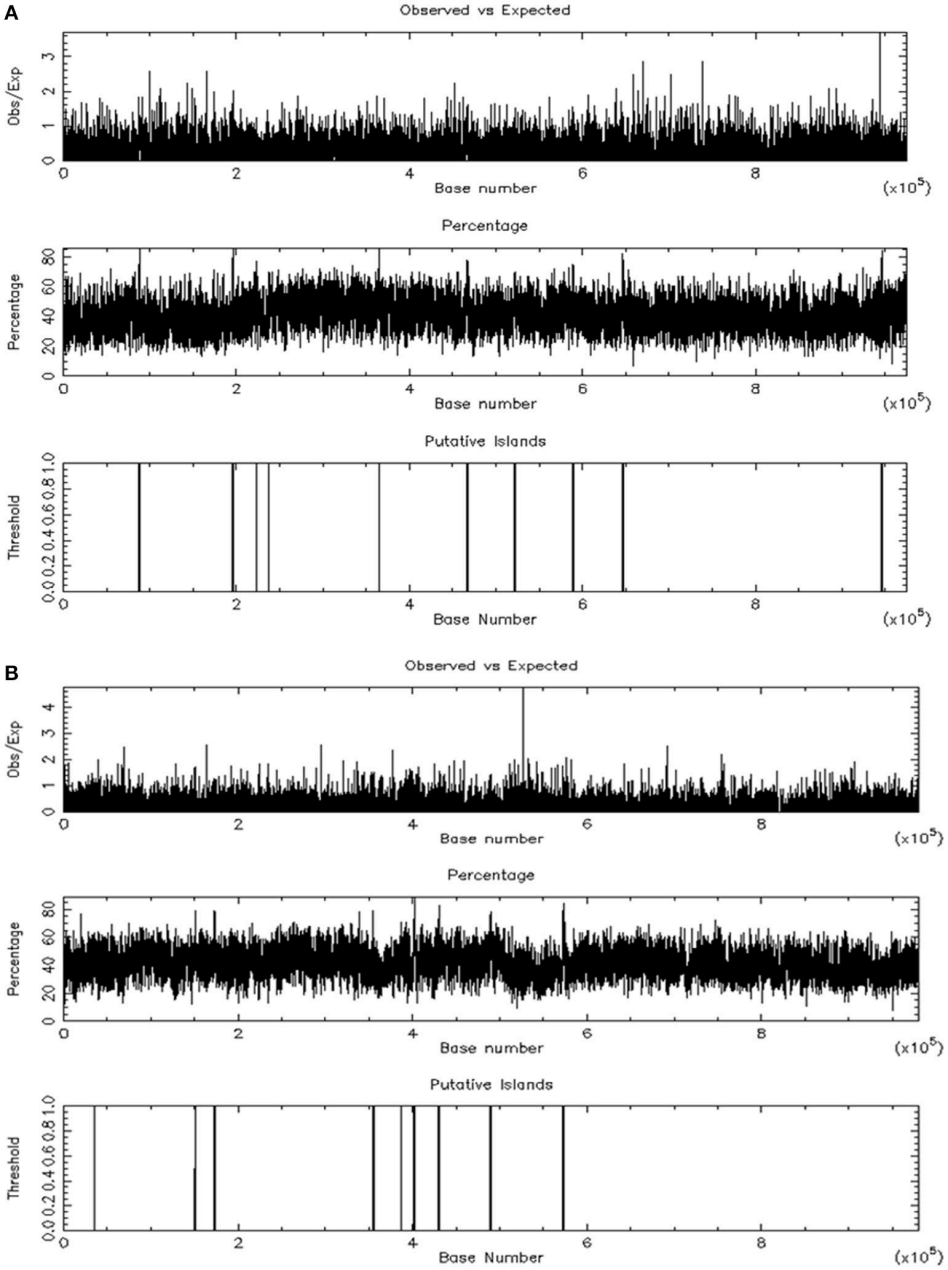
CpG islands identified around two significant SNPs on two chromosomes. The SNP is located in the center of the sequence on chromosome 6 **(A)** and on chromosome 7 **(B)**.

CpG Islands can be predicted by searching for CpG repeats with a CG content of at least 50%. The sequence length ranges from 200 bp to several Mbp. We screened CpG islands within 0.5 Mbp (upstream or downstream) of each significant SNP. A number of CpG islands were identified close to the SNPs on chromosomes 1, 6, and 7 (listed in Table S9 and illustrated for chromosomes 6 and 7 in Figures 6A,B, respectively). Interestingly, the *KCNQ1OT1* lncRNA and the CpG islands on chromosome 6 are close enough to interact (based on orthologous lncRNAs in other species).

#### TF binding sites

Some genetic variants or epigenetic regulations may affect TF binding sites. First, all the TF binding sites on the equine genome were predicted using bioinformatics pipelines and TF databases. We then extracted a list of TFs located within 1 Mbp (upstream or downstream) of the SNPs on chromosomes 1, 6, 7, 16, and 29 (Table S10). The number of predicted binding sites and the number of TF candidates are indicated in Table 3. Chromosome 29 presented 17 binding sites and 134 potential TFs within 1 Mbp upstream or downstream of the SNP. Two of these TFs were involved in muscle maintenance (muscle initiator and MyoD) and others were involved in mitochondrial biogenesis (NF1 and PPARγ). On chromosome 16, we identified 11 binding sites and 34 putative TFs (again including PPARγ, directly involved in mitochondrial biogenesis).

## Discussion

The results of the present GWAS show how a complex trait like endurance exercise ability is determined by a range of genes, regulatory loci and (probably) epigenetic regulations. None of the five QTL significantly associated with performance explained more than 1.3% of the variance for each trait. The heritability (h^2^) of endurance riding performance has been estimated to range from 0.20 to 0.28 for average race speed (Table S1; Ricard and Touvais, 2007). Hence, the present GWAS results revealed only a small proportion of the genetic variants that influence endurance ability. Some genetic variants with small effects can be detected with a frequency of more than 5% in the population. In contrast, many very low-frequency variants with small or moderate effects would not have been detected in our GWAS due to the small number of observations (McCarthy and Hirschhorn, 2008; Manolio et al., 2009). In such a case, the number of observations and the genetic structure of the population determine the power of the study. In fact, we studied a population of horses with a favorable genetic structure and a mean performance index just above the national average. The LD between nearby SNPs (0.26 at a mean distance between adjacent SNPs of 39.8 kb) was similar to those reported for species in which QTL have been found. McKay et al. (2007) studied LD in five bovine breeds and found a mean *r*^2^ of 0.50 at 5 kb and 0.22 at 199 kb, which is similar to our present finding. The values found in sheep differed more, with *r*^2^ values of between 0.12 and 0.19 at 50 kb (Kemper et al., 2011). However, the low number of horses in the present sample prevented us from studying complex polygenic traits. Model 1 had a power of 98%, with a type I error rate of 5%, an SNP effect of 0.25 phenotypic SDs, and a frequency of 50%. Depending on the LD, this corresponds to a QTL effect of 0.45 phenotypic SDs at the midpoint between two adjacent SNPs. However, when considering the 597 horses with performance traits, the power was only 71% with a frequency of 10%. Thus, only strong effects could have been detected. Given the high likelihood of polygenic determinism for complex performance traits in endurance riding, we used three complementary models to take account of genetic backgrounds with different dominance patterns. The single allele effect in model 1 was best suited to detecting additive effects. The genotype effect in model 2 is better to identifying strong dominance. Lastly, the haplotype model 3 was best suited to detecting trait-associated clusters of genes or loci distributed across a large portion of the chromosome.

In view of the rather high heritability for performance traits in endurance riding (Ricard and Touvais, 2007), we expected to find important QTL marked by significant SNPs. However, we observed five significant QTL#6, 1, 7, 16, and 29 distributed over five corresponding chromosomes, rather than strong quantitative trait loci. Surprisingly, the QTL were more strongly associated with the race distance and finishing status, which are less heritable criteria (0.10) than the average race speed. This result shows that endurance ability is a complex trait governed by a group of genes and regulatory loci, rather than by a single major QTL. However, we found two intronic SNPs in the *SORCS3* and *SLC39A12* genes, which respectively accounted for only 1.1 and 0.86% of the total phenotypic variance.

The *SORCS3* gene codes for a transmembrane receptor from the vacuolar protein sorting (VPS) 10 family, which also includes *SORT1, SORL1, SORCS1*, and *SORCS2*. These receptors interact with the retromer protein complex, and have pleiotropic functions in endosomal, lysosomal, and external trafficking. *SORCS3* is thought to have a role in type I and II diabetes via an interaction with the insulin-sensitive glucose transporter *GLUT4* (Lane et al., 2012). Expression of this gene is induced by neuronal activity in the hippocampus (Hermey et al., 2004), and thus it has been suggested that *SORCS3* is indirectly involved in synaptic plasticity (Hermey et al., 2013). In the mouse, functional knockdown of SORCS3 increases amyloid precursor protein processing (Reitz et al., 2013), and the animals display reduced synaptic transmission, long-term depression, impaired spatial learning and increased fear extinction (Breiderhoff et al., 2013). *SORCS3* and other VPS10 receptor family members are involved in neurotrophin pathways such as the neuronal growth factor pathway (Westergaard et al., 2005). The relationship between these neuronal functions and endurance exercise activity might be related to the neuroprotective role of endosomal trafficking and the neurotrophin pathway with regard to the cell stress caused by hypoxia, reactive oxygen species production, endotoxin circulation and massive proteolysis related to long-lasting, intense exercise. Interestingly, blood expression levels of genes coding for the retromer complex (*SORT1, SNX3, SNX5, SNX10, SNX20*, and *SNX24*) that interacts with VPS10 family receptors were elevated after an 8-h, 160 km endurance ride (Mach et al., 2016). Thus, the endosomal trafficking pathway in neurons and other cells is probably upregulated during endurance exercise, and some transcripts may be released into the circulation.

*SORCS1* is located close to the *SORCS3* locus, and belongs to the same protein family. It has been identified as a QTL for type II diabetes in rat and mice (Clee et al., 2006; Granhall et al., 2006) and type I diabetes in humans (Paterson et al., 2010). The sortilin 1 protein (*SORT1*) interacts with *SORCS* family members in endosomal trafficking, and has an important role in the trafficking of insulin-responsive vesicles containing the glucose transporter Glut4 (Jedrychowski et al., 2010). Glycemia homeostasis and intracellular transport are critical mechanisms in exercise-related muscle and neuron metabolism.

We found an intronic SNP in the *SLC39A12* gene on chromosome 29 (QTL#29). The *SLC39A12/*ZIP-12 protein has a high affinity for zinc and belongs to the ZIP zinc transporter family. ZIP-12 imports zinc from the extracellular space and/or transfers it to intracellular compartments. Zinc has many critical roles as an enzyme cofactor for more than 1,000 proteins involved in DNA repair, epigenetic regulation, cell signaling and catalysis. The ZIP12 protein encoded by *SLC39A12* is known to have a role in neuronal structure and function; (Chowanadisai et al., 2013). It is highly expressed in the brain (and especially in the hippocampus) and has critical function in neuronal embryo development and neuronal differentiation.

It is noteworthy that we identified significant variants of two candidate genes involved in neuronal function in general and the central nervous system (CNS) in particular. In an exercise activity like endurance riding, neuronal/CNS contributions to performance may be very important because elite athletes have a high threshold for the conscious and unconscious perception of peripheral and central fatigue. This enables them to perform beyond the normal physiological threshold for injury (Noakes, 2012). The recently proposed “central governor” model highlights the importance of the CNS in sporting performance; many elite athletes and riders report that mental factors are critical in this respect.

Interestingly, ZIP12 has a recently discovered regulatory role in hypoxia-induced pulmonary hypertension (Zhao et al., 2015). Endurance exercise is an intense aerobic activity, and the horse's lung is subjected to alveolar hypoxia at submaximal running speeds and may even bleed at high speeds (Sullivan and Hinchcliff, 2015). After chronic exposure to hypoxia, ZIP12 is overexpressed in pulmonary vascular smooth muscles and endothelial cells in rats, cattle and humans; this shows that ZIP12 upregulation in the pulmonary vasculature is a common response to chronic hypoxia. In the horse, hypoxemia is even observed during moderate exercise (60% of VO_2_max) and is associated with hypercapnia at high intensity (Bayly et al., 1983; Wagner et al., 1989). Impaired gas exchange could be mainly due to poor alveolar-capillary diffusion (60%) and ventilation/perfusion mismatch (40%) (Nyman et al., 1995). In view of all these findings, one can hypothesize that a combination of the two intronic SNPs in *SLC39A12* (BIEC_755603 and BIEC_755604) produces a ZIP12 variant that is less sensitive to the chronic alveolar hypoxia caused by critical ventilation during exercise, and thus facilitates alveolar-capillary gas diffusion. Furthermore, 17 loci for TF binding sites (134) are predicted in the region 1 Mbp downstream of the *SLC39A12* gene on chromosome 29, and some of these may be related to ZIP12 transcription.

As in many GWAS, we found three intergenic SNP (QTL#6, 7, and 16): these may be markers of key genomic regulatory functions in response to the stress of endurance exercise. On chromosome 6, the SNP BIEC_1022884 was associated with distance and finishing status (according to three different polygenic models). The intergenic sequence pointed out by this QTL#6 was predicted to include a lncRNA ortholog of *KCNQ1OT1*, which is already known to be involved in long-range epigenetic regulation in *cis* and *trans* in the human and the mouse (Mohammad et al., 2008). A growing number of lncRNAs have been predicted (up to 15,000) and their various regulatory functions are progressively identified. The lncRNA's function (but not its full sequence) is usually conserved among species. However, bioinformatics databases and pipelines enable the comparison of the sequences' respective molecular structures and the prediction of candidates such as *KCNQ1OT1* lncRNA (which was partially aligned with the horse genome and the genomes of other domesticated animals). Surprisingly, we identified this lncRNA on chromosome 6 (where the QTL#6 was located) but not on the chromosome 12 (where the *KCNQ1* gene has been annotated in the reference genome EquCab2). Furthermore, we validated this *in silico* prediction by detecting copies of the candidate lncRNA in total RNA extracted from equine culture cells (myoblasts and fibroblasts). The human *KCNQ1OT1* lncRNA is a 91kb antisense transcript expressed from intron 10 of the Kcnq1 gene on the paternal chromosome. From a molecular point of view, this lncRNA is known to interact with CpG islands to recruit histone methylase and thus to inactivate genes by imprinting the loci. In humans and mice, *KCNQ1OT1* transcribed from the paternal chromosome has a bidirectional silencing effect on many genes in the Kcnq1 cluster located on the maternal chromosome, where it modifies the chromatin structure and thus the transcription of upstream or downstream genes. Direct epigenetic regulation by the *KCNQ1OT1* lncRNA has been demonstrated in the placenta; the histones H3K9 and H3K27 specifically interact with chromatin via the Kcnq1 domain (Pandey et al., 2008). Alterations in this region and subsequent anomalies in imprinting regulation have been reported in several human diseases, (Table S11; Chen et al., 2013). Taken as a whole, the literature data and our present results show that *KCNQ1OT1* is a good candidate lncRNA in horse and genetic, and that variations in this RNA may affect its epigenetic functions via different mechanisms (RNA-chromatin interactions, histones methylation modifications, etc.). However, its putative regulatory functions in exercise-related pathways in the horse must now be extensively investigated. Mutations in KCNQ1 have been identified in human patients with severe ventricular arrhythmia, a long QT interval and slow ventricular repolarization on the ECG signal (Wu et al., 2016; Maltese et al., 2017). If the lncRNA were to modulate the expression of the *KNCQ1* gene (and perhaps other genes) by hybridization and the induction of conformational changes in the chromatin, cardiac excitability might be affected. Interestingly, a quick cardiac recovery (i.e., short recovery time to reach 64 bpm after the race) is an important performance factor for passing the veterinary examination after each phase of equine endurance events (Younes et al., 2015).

Lastly, it is noteworthy that six significant CpG islands were found within 10685-40770 bp around the QTL#6. Thus, some of these CpG islands may interact with the candidate *KCNQ1OT1* lncRNA.

MiRNAs (small non-coding RNAs 18-24 nt) are responsible for post-transcription regulation via RNA interference with the assembly of the RISC complex after a specific maturation pathway. Four miRNAs were identified within 1 to 3.35 Mbp of the SNP's location on chromosomes 6, 16, 1, and 7. Some miRNAs (such as eca-miR-26a) regulate protein translation by RNA interference (by cleavage or inhibition of translation) for sets of up to several 100 genes. Among the enriched pathways putatively regulated by the four miRNAs, the following are directly related to exercise: regulation of the actin cytoskeleton (related to cell trafficking, motion and structure), circadian rhythm (related to energetics and hormonal regulation), glycosaminoglycan biosynthesis (related to joint function), protein processing endoplasmic reticulum (related to the exercise recovery and homeostasis), and ubiquitin mediated proteolysis (related to the cell and metabolic stress and massive proteolysis caused by long endurance exercise).

We explored potential regions involved in epigenetic regulation via the methylation of cytosine, which influences the structure of the chromatin in many ways. In mammalian genomes, CpG islands are typically 300–3,000 bp in length, and have been found in or around approximately 40% of gene promoter regions. Indeed, about 70% of human promoters have a high CpG content. In the present study, we found many CpG islands within 10–40 kb of the SNPs on chromosomes 1, 6, and 7. On chromosomes 1 and 6, these CpG islands might be close to the promoters of the candidate genes previously identified in these regions: *SORCS3* (QTL#1) and *SLC39A12* (QTL#29). The QTL#6 contains a range of putative non-coding sites (the *KCNQ1OT1* lncRNA and CpG islands) that may interact and thus influence performance traits such as distance and finishing status. This lncRNA might interact with chromatin, histones, CpG islands and gene clusters, as has been demonstrated for many other lncRNAs (Guttman et al., 2009).

Lastly, there are many TF binding sites in the genome, and some can be predicted with bioinformatics tools. A mutation within the site can affect the binding affinity for the specific TF and thus modify the entire pathway. Again, epigenetic regulation (by changes in the chromatin structure) can also affect TF accessibility. We predicted binding site loci on chromosomes 29, 16 and (to a lesser extent) 6. Each binding site has many predicted candidate TFs. Several well-known candidates are related to exercise, including PPARγ and NF1 on chromosome 29, PPARγ on chromosome 16 (all of which contribute to activation of the mitochondrial biogenesis pathway by repeated bouts of training), and MyoD, Oct4 and muscle initiator on chromosome 29 (which are involved in the proliferation of satellite cells and the latter's differentiation into myoblasts after partial, physiological rhabdomyolysis during endurance exercise). NF-κB on chromosome 6 is predicted to be involved in inflammation signaling. This might be related to the high inflammation response and catabolism observed in muscle tissue and systemically after a 120–160 km ride (Barrey et al., 2006; Capomaccio et al., 2010; Mach et al., 2016).

## Conclusion

Our GWAS identified five significant QTL associated with the endurance performance traits (distance, finish status and average speed) in Arabian horses. This demonstrates the polygenic nature of these complex traits. These five QTL were located variously on chromosomes 1, 6, 7, 16, and 29 and explains between 0.43 and 1.29% of the trait variance. The QTL#6 is the most significant hit; we predicted and (using RT-qPCR) detected a new candidate equine lncRNA ortholog of *KCNQ1OT1* which might have a regulatory function in the cardiac recovery (QC wave lengthening = slow down heart rate). QTL#1 and 29 were defined by two intronic SNPs in well-conserved genes (*SORCS3* and *SLC39A12*) with known pleiotropic cellular functions in neuronal tissues (Central nervous system CNS). One can legitimately hypothesize that these two QTL#1 and 29 have critical roles in neuronal functions during exercise. The “winner's mentality” is often cited by elite athletes and high-level trainers but is still poorly understood by the scientists. The same is true of the elite horses (and indeed riders) in equestrian sports; in fact, the best horses are often described as being highly motivated and tenacious. The predicted annotations of QTL# 7 and 16 might be related to other regulatory elements such as miRNAs, TF biding sites, CpG islands and CNV variants. These genomic elements might contribute to the adaptation to endurance exercise via the indirect regulation of several pathways such as cell trafficking, zinc homeostasis, mitochondrial metabolism and biogenesis, cytoskeletal proteins and glycosaminoglycan biosynthesis. Taken as a whole, the present study demonstrates that endurance performance is a complex physiological trait with polygenic determinism.

## Author contributions

AR: Contributed to the experimental design of the study, performed the statistical models and GWAS analysis, and wrote the paper. CR: Contributed to the experimental design, collected the blood samples, and wrote the paper. CB: Data management for performance records, conformation measurements, and pedigree files. FB: Contributed to the GWAS model computation. GT: Contributed to the KCNQ1OT1 lncRNA candidate detection by RT-qPCR on equine culture cells. NM: Contributed to data collection and bioinformatics (data mining). CM: Data collection and data mining. JR: Data collection, blood sample. XM: Data collection, blood sample collection and genotyping management. LS: Contributed to the experimental design, data collection and genotyping. EB: Managed the project, experimental design, data collection and data mining, and wrote the paper.

### Conflict of interest statement

The authors declare that the research was conducted in the absence of any commercial or financial relationships that could be construed as a potential conflict of interest.
