# Supplementary material for: Endurance Exercise Ability in the Horse: A Trait with Complex Polygenic Determinism
Source: Front Genet. 2017 Jun 28;8:89. doi: 10.3389/fgene.2017.00089 (PMC5488500; doi:10.3389/fgene.2017.00089)
Supplement: Supplementary file 2 [file Table2.pdf]

SUPPLEMENTARY MATERIAL:

Title: Endurance exercise ability in the horse: a trait with complex polygenic determinism

Anne Ricard[1,2§], Céline Robert [1,3], Christine Blouin, Fanny Baste[1], Gwendoline Torquet [1], Caroline Morgenthaler[1], Julie Rivière[1], Nuria Mach [1], Xavier Mata[1], Laurent Schibler[1], Eric Barrey[1§]
[§] corresponding authors

Figure S1: Details on the twelve haplotypes associated to the averaged speed. Frequency of twelve significant haplotypes centred on SNP BIEC_11782. The haplotypes are ordered by decreasing frequency. Among the 4 most frequent haplotypes, 2 are negatively associated with the speed and 2 are posivlely associated with the speed but less frequent. The last 6 haplotypes are significant but their effect was poorly estimated. Each haplotype was define by a set of 7 SNP centred on SNP BIEC_11782 (Chr1: 25715334). File Image 1.PDF

Figure S2: Genetic map of *SORCS3* gene on equine chromosome 1. One intronic SNP BIEC2_11782 was detected in intron #16. This SNP was significantly associated with the average race speed in endurance ride. File Image 2.PDF

Figure S3: Genetic map of *SLC39A12* gene on equine chromosome 29. Two close SNPs BIEC2_75563 and BIEC2_755604 were detected in intron #51. These SNP was significantly associated with the finishing status in endurance ride. File Image 3.PDF

Table S1: Phenotypes and pseudo-performance values of the 597 horses genotyped. This table presents the three traits as pseudo-performance and the equivalent number of races performed by the horse (ENP). The pseudo-performance value was weighted by the number of observations per horse and by genetic parameters. The weighting factor was referred to as the equivalent number of performances (ENP). All the horses are Purebred Arabian or crossed Arabian with at least 50% Arabian blood (see the breed codes used by the French studbook (SIRE) in the panel 2). Performances were measured in French endurance races from 2002 to 2011 (38473 results, 7363 horses) with 3 traits: speed (S; standardized by race), total run distance (D), Finishing status (F, discrete measure: qualified/retired/eliminated). According to Ricard et Touvais (2007) and after updating with more recent genetic parameters, the following multitrait model was used:

$$\mathbf{y} = \mathbf{Xb} + \mathbf{Zu} + \mathbf{Zp} + \mathbf{e}$$

With **y** the vector of performances for the 3 criteria, $\mathbf{b}$ vector of fixed effect, which included sex (female, male, castrated), age (form 6 years to 12 years by 1 and 13 years and more), race (2263 races, no race effect for the trait D), $\mathbf{p}$ the vector of permanent environmental random effect common to the different performances of the same horse (7363 effects for each trait), and $\mathbf{a}$ the random polygenic effect (35068 horses including ancestors for each trait). **X** and **Z** are incidence matrices. The variance-covariance matrices of random effects were:

$V(\mathbf{a}) = \mathbf{A} \otimes \mathbf{G}$ with $\otimes$ the direct product, $\mathbf{A}$ the relationship matrix and $\mathbf{G} = \begin{bmatrix} \sigma^2_{a,1} & \sigma_{a,12} & \sigma_{a,13} \\ \sigma_{a,12} & \sigma^2_{a,2} & \sigma_{a,23} \\ \sigma_{a,13} & \sigma_{a,23} & \sigma^2_{a,3} \end{bmatrix}$ the genetic variance and covariance between the 3 traits (1=S, 2=D and 3=F), $V(\mathbf{p}) = \mathbf{I} \otimes \mathbf{P}$ and $\mathbf{P} = \begin{bmatrix} \sigma^2_{p,1} & \sigma_{p,12} & \sigma_{p,13} \\ \sigma_{p,12} & \sigma^2_{p,2} & \sigma_{p,23} \\ \sigma_{p,13} & \sigma_{p,23} & \sigma^2_{p,3} \end{bmatrix}$ the permanent environmental variance and covariance between the 3 criteria

and $V(\mathbf{e}) = \mathbf{I} \otimes \mathbf{P}$ and $\mathbf{R} = \begin{bmatrix} \sigma^2_{e,1} & 0 & 0 \\ 0 & \sigma^2_{e,2} & 0 \\ 0 & 0 & \sigma^2_{e,3} \end{bmatrix}$. We used Gibbs sampling (Software TM, [2]) to estimate the genetic parameters defined as: the heritability $h_i^2 = \frac{\sigma^2_{a,ii}}{\sigma^2_{a,ii} + \sigma^2_{p,ii} + \sigma^2_{e,ii}}$ and the repeatability $r_i = \frac{\sigma^2_{a,ii} + \sigma^2_{p,ii}}{\sigma^2_{a,ii} + \sigma^2_{p,ii} + \sigma^2_{e,ii}}$ for i=1,2,3, the genetic correlation $r_{a,ij} = \frac{\sigma_{a,ij}}{\sigma_{a,ii}\sigma_{a,jj}}$, the correlation between horse abilities ($a_{i,k} + p_{i,k}$ for the horse *k* and trait *i*) $r_{h,ij} = \frac{\sigma_{a,ij} + \sigma_{p,ij}}{\sqrt{(\sigma^2_{a,ii} + \sigma^2_{p,ii})(\sigma^2_{a,jj} + \sigma^2_{p,jj})}}$ for i=1,2,3 and j=1,2,3 .

They are summarized in Table 1. For GWAS analysis, de-regressed estimates of horse ability ($a_{i,k} + p_{i,k}$ for the horse *k* and trait *i*) of genotyped horses were used. The pseudo-performance was then:

$y^*_{i,k} = \frac{\hat{a}_{i,k} + \hat{p}_{i,k}}{\rho_{i,k}}$ for trait *i* and horse *k* with $\rho_{i,k}$ the reliability of the sum of estimates $\hat{a}_{i,k} + \hat{p}_{i,k}$ obtained with the previous model. In that case, the residual variance associated to this pseudo-performance in a mixed model with polygenic effect is $V(e_{i,k}) = \frac{1}{m_{i,k}} \sigma^2_{e_i}$ with $m_{i,k} = \frac{\rho_{i,k}(1-r_i)}{(r_i - \rho_{i,k} h_i^2)}$. The pseudo-performance $y^*_{i,k}$ may be considered as a corrected mean of performances for fixed effects and the variable $m_{i,k}$ as an equivalent number of performances (ENP) used to calculate this mean. Estimated horse abilities (EHA) of the sample compared to whole population are given Table S2, as well as pseudo-performances and their equivalent number of performances for the sample in Table S3. Horses used in GWAS are a selected sample as mean of EHA are higher than overall mean (about half standard deviation) but moderately and with a maintained diversity (same variance of EHA).
File Table 1.XLSX

Table S2: Elementary statistics of estimated horse ability (EHA, breeding value plus environmental effect) in endurance races for all horses (n=7363) and for sample used in GWAS (n=597) in unit of phenotypic standard deviation.

Traits	Mean		SD		Min.		Max.	
	All	Genotyped	All	Genotyped	All	Genotyped	All	Genotyped
EHA Speed	0.18	0.36	0.48	0.52	-1.23	-1.12	1.73	1.69
EHA Distance	0.15	0.37	0.36	0.38	-0.76	-0.56	1.61	1.48
EHA Finishing status	0.13	0.33	0.34	0.36	-0.80	-0.53	1.43	1.36
Reliability Speed	0.57	0.69	0.22	0.15	0.00	0.09	0.93	0.92
Reliability Distance	0.59	0.68	0.17	0.14	0.00	0.17	0.92	0.90
Reliability Finishing status	0.56	0.64	0.16	0.13	0.03	0.15	0.89	0.87

Table S3: De-regressed estimated ability (breeding value plus environmental effect), used as the pseudo-performance indicator in the GWAS (in phenotypic SD units) and the equivalent number of performances (ENP) (n=597).

Traits	Mean	SD	Minimum	Maximum
Speed	0.51	0.76	-1.81	3.15
Distance	0.54	0.55	-1.01	2.12
Finishing status	0.51	0.55	-0.98	1.99
ENP Speed	1.65	0.51	0.13	2.60
ENP Distance	4.69	1.32	0.80	7.31
ENP Finishing status	4.72	1.37	0.72	7.61

Table S4: list of the three pairs of primers designed to detect the expression of the equine lncRNA candidate *KCNQ1OT1* orthologue. The endogenous gene used was 18S**.**

Primer's ID	Sequence (5' -3')	Position within target sequence	Amplicon length
KCNQ1OT1-P1-FWD	GCTTGCTTTGCTTTTACTC	182-200	194
KCNQ1OT1-P1-REV	GTTCTTTCTTGTTCTGTGTC	356-375	
KCNQ1OT1-P2-FWD	GCCTTGTTTTTTTCTCTGCT	829-848	222
KCNQ1OT1-P2-REV	TCGTTCTCTGCCTCTTCC	1033-1050	
KCNQ1OT1-P3-FWD	TGGTCACCTCCCATAACT	1967-1984	234
KCNQ1OT1-P3-REV	TTGTCCCTCTACCCTTCT	2183-2200	
**Endogenous gene**			
18S-FWD	AGGGTTCGTTCCTGTGTTCC	975-994	92
18S-REV	GGCCACGCTTCCTTACAGAT	1066-1047	

Table S5: List of all the annotated genes located 4 Mbp upstream and 4 Mbp downstream of the SNPs associated with the performance traits. File   Table 5.XLSX

Table S6: List of miRNAs located within 1Mpb of the SNP associated with performance traits.

QTL#	SNP	Distance / SNP	miRNA name	5' position	# Gene targets	# Pathways
6	BIEC2_1022884	2 107 650	eca-mir-763-5p	81 420 420	465	NA
1	BIEC2_11782	2 001 212	eca-miR-146b-5p & -3p	27716546	863	140
7	BIEC2_977605	3 350 550	eca-miR-7-2-5p	2 932 716	1218	156
16	BIEC2_336016	1 312 983	eca-mir-1289-3p	27 474 897	262	6

Table S7: Predicted pathways and number of genes targeted by the miRNAs 146b and 1289 (located close to the SNPs associated with performance traits).

KEGG pathway	p-value	#genes	#miRNAs
Wnt signaling pathway	8.79E-12	36	4
Gap junction	3.48E-09	19	4
Neurotrophin signaling pathway	1.19E-08	27	4
Pathways in cancer	1.19E-08	58	4
Long-term potentiation	6.42E-08	18	4
ErbB signaling pathway	8.45E-08	22	4
Circadian rhythm	3.14E-07	10	3
Ubiquitin mediated proteolysis	3.58E-06	27	3
Axon guidance	7.67E-06	26	4
TGF-beta signaling pathway	2.02E-05	18	4
Glycosaminoglycan biosynthesis - chondroitin sulfate	2.38E-05	3	2
Focal adhesion	2.46E-05	34	4
Dopaminergic synapse	2.97E-05	24	4
Hedgehog signaling pathway	3.21E-05	12	3
GnRH signaling pathway	4.90E-05	18	4
Adherens junction	5.71E-05	17	3
Biotin metabolism	0.000137576	1	1
mTOR signaling pathway	0.000144749	14	2
Regulation of actin cytoskeleton	0.000219436	34	4
Amphetamine addiction	0.000308041	15	4
Protein processing in endoplasmic reticulum	0.000337889	28	4
MAPK signaling pathway	0.000544857	40	4
Insulin signaling pathway	0.000610997	23	4
Fc gamma R-mediated phagocytosis	0.001155323	17	4
Melanogenesis	0.001155323	18	4
VEGF signaling pathway	0.001165635	13	4
Notch signaling pathway	0.001439462	10	2
Lysine degradation	0.003811159	10	4
PI3K-Akt signaling pathway	0.01124231	43	4
p53 signaling pathway	0.01366734	12	4
Long-term depression	0.0323868	13	4
Serotonergic synapse	0.03896708	18	3
Tight junction	0.03896708	20	4

Table S8: Results of the RTqPCR after reverse transcription of total RNA extracted from primary cultures of equine myoblasts and fibroblasts.

**Cell**	**mean Ct**	**SD Ct**	**$2^{-\Delta\Delta Ct}$**
Fibrobasts	29.43	1.01	1.00
Myoblasts	30.23	3.99	0.57

Table S9: CpG islands found near to the significant SNPs.

QTL #	Distance from SNP	ID on the sequence	CpG start	CpG end
6	20 369	ID=EMBOSS_001.13	520379	520999
	21 251	ID=EMBOSS_001.14	521261	521871
	32 778	ID=EMBOSS_001.12	467232	467690
	33 258	ID=EMBOSS_001.11	466752	467224
	33 880	ID=EMBOSS_001.10	466130	466513
	34 357	ID=EMBOSS_001.9	465653	466004
1	38 556	ID=EMBOSS_001.2	538566	538874
	39 385	ID=EMBOSS_001.3	539395	539671
	39 673	ID=EMBOSS_001.4	539683	540015
	40 275	ID=EMBOSS_001.5	540285	540734
	40 770	ID=EMBOSS_001.6	540780	541136
7	10 685	ID=EMBOSS_001.13	489325	489615
	11 074	ID=EMBOSS_001.12	488936	489294

Table S10: List of TF binding sites and candidate TFs located close to the QTL. File Table 10.XLSX

Table S11: Annotations of transcriptomic disorders and diseases with *KCNQ1OT1* deregulation in humans and in mouse models (extracted from the LncRNA database). File Table 11 XLSX
