# Supplementary material for: Endurance Exercise Ability in the Horse: A Trait with Complex Polygenic Determinism
Source: Front Genet. 2017 Jun 28;8:89. doi: 10.3389/fgene.2017.00089 (PMC5488500; doi:10.3389/fgene.2017.00089)
Supplement: Supplementary file 5 [file Table11.PDF]

The LncRNA and Disease Database

Home | Browse | Search | Interaction | Predict | Download | Submit
Help

You can search the entries by such keywords:

LncRNA kcnq1 **Click to Search**

Reset all

LncRNA name	Disease name	Dysfunction type	Description	Chr	Start	End	Strand	Species	Alias	Genbank	Sequence	Reference
KCNQ1DN	aging	Epigenetics	Continuously hypermethylated upon aging.	chr11	2891263	2893335	+	Human	KCNQ1DN; BWRT; HSA404617	NR_024627.1	Gene / RNA	22067257
KCNQ1DN	Wilms' tumor	Expression	A novel imprinted gene, KCNQ1DN, within the WT2 critical region of human chromosome 11p15.5 and its reduced expression in Wilms' tumors.	chr11	2891263	2893335	+	Human	KCNQ1DN; BWRT; HSA404617	NR_024627.1	Gene / RNA	11056398
KCNQ1OT1	Beckwith-Wiedemann syndrome	Epigenetics	In Beckwith-Wiedemann syndrome (BWS), approximately 50% of patients show loss of DNA methylation accompanied by loss of histone H3 Lys9 dimethylation on maternal KCNQ1OT-DMR, namely an imprinting disruption, leading to diminished expression of CDKN1C.	chr11	2661768	2721228	-	Human	KCNQ1OT1; LIT1; KvDMR1; KCNQ10T1; KCNQ1-AS2; KvLQT1-AS; NCRNA00012	NR_002728.3	Gene / RNA	16575194
KCNQ1OT1	Beckwith-Wiedemann syndrome	Epigenetics	The 5'end of the KCNQ1OT1 gene is hypomethylated in the Beckwith-Wiedemann syndrome.	chr11	2661768	2721228	-	Human	KCNQ1OT1; LIT1; KvDMR1; KCNQ10T1; KCNQ1-AS2; KvLQT1-AS; NCRNA00015		Gene / RNA	12136243
KCNQ1OT1	Beckwith-Wiedemann syndrome	Locus	In the human and mouse BWS imprinting regions, two major elements for regulation of imprinted gene expression have been identified athe imprinting centers IC1 and IC2.IC2 appears to be the promoter of the paternally expressed probably noncoding transcript.	chr11	2661768	2721228	-	Human	KCNQ1OT1; LIT1; KvDMR1; KCNQ10T1; KCNQ1-AS2; KvLQT1-AS; NCRNA00018	NR_002728.9	Gene / RNA	15590939
KCNQ1OT1	Beckwith-Wiedemann syndrome	Locus	KvDMR1 and/or its associated antisense RNA (KvLQT1-AS) represents an additional imprinting control element or center in the human 11p15.5 and mouse distal 7 imprinted domains.	chr11	2661768	2721228	-	Human	KCNQ1OT1; LIT1; KvDMR1; KCNQ10T1; KCNQ1-AS2; KvLQT1-AS; NCRNA00013	NR_002728.4	Gene / RNA	10393948
KCNQ1OT1	Beckwith-Wiedemann syndrome	Locus	The LIT1 CpG island can act as a negative regulator in cis for coordinate imprinting at the centromeric domain, thereby suggesting a role for the LIT1 locus in a BWS pathway leading to functional inactivation of p57(KIP2).	chr11	2661768	2721228	-	Human	KCNQ1OT1; LIT1; KvDMR1; KCNQ10T1; KCNQ1-AS2; KvLQT1-AS; NCRNA00014		Gene / RNA	10958646
KCNQ1OT1	Beckwith-Wiedemann syndrome	N/A	In vitro fertilization may increase the risk of Beckwith-Wiedemann syndrome related to the abnormal imprinting of the KCN1OT gene.	chr11	2661768	2721228	-	Human	KCNQ1OT1; LIT1; KvDMR1; KCNQ10T1; KCNQ1-AS2; KvLQT1-AS; NCRNA00019	NR_002728.10	Gene / RNA	12746837
KCNQ1OT1	Beckwith-Wiedemann syndrome	N/A	LIT1 (KCNQ1OT1) may play a role in Beckwith-Wiedemann syndrome.	chr11	2661768	2721228	-	Human	KCNQ1OT1; LIT1; KvDMR1; KCNQ10T1; KCNQ1-AS2; KvLQT1-AS; NCRNA00017		Gene / RNA	15888726
KCNQ1OT1	Beckwith-Wiedemann syndrome	regulation	Epigenetic deregulation of lncRNAs genes is associated with disease	chr11	2661768	2721228	-	Human	KCNQ1OT1; LIT1; KvDMR1; KCNQ10T1; KCNQ1-AS2;	NR_002728.3	Gene / RNA	23791884

									KvLQT1-AS; NCRNA00012			
KCNQ1OT1	colorectal cancer	Epigenetics	epigenetic disruption	chr11	2661768	2721228	-	Human	KCNQ1OT1; LIT1; KvDMR1; KCNQ10T1; KCNQ1-AS2; KvLQT1-AS; NCRNA00016		Gene / RNA	16965397
KCNQ1OT1	colorectal cancer	expression	Recent studies have linked their mis-expression to diverse cancers (ANRIL: prostate cancer, XIST: female cancers, HOTAIR: breast cancer, KCNQ1OT4: colorectal cancer).	chr11	2661768	2721228	-	Human	KCNQ1OT1; LIT1; KvDMR1; KCNQ10T1; KCNQ1-AS2; KvLQT1-AS; NCRNA00012	N/A	Gene / RNA	23660942
KCNQ1OT1	hepatocelluar carcinoma	mutation	A novel tetranucleotide repeat polymorphism within KCNQ1OT1 confers risk for hepatocellular carcinoma.	chr11	2661768	2721228	-	Human	KCNQ1OT1; LIT1; KvDMR1; KCNQ10T1; KCNQ1-AS2; KvLQT1-AS; NCRNA00012	NR_002728.3	Gene / RNA	23984860
KCNQ1OT1	kidney cancer	regulation	Oncogene	chr11	2661768	2721228	-	Human	KCNQ1OT1; LIT1; KvDMR1; KCNQ10T1; KCNQ1-AS2; KvLQT1-AS; NCRNA00012	NR_002728.3	Gene / RNA	24373479

Citation: Chen et al., LncRNADisease: a database for long-non-coding RNA-associated diseases. Nucleic Acids Res. 2013 Jan 1;41(D1):D983-6.
