# Supplementary material for: Endurance Exercise Ability in the Horse: A Trait with Complex Polygenic Determinism
Source: Front Genet. 2017 Jun 28;8:89. doi: 10.3389/fgene.2017.00089 (PMC5488500; doi:10.3389/fgene.2017.00089)
Supplement: Supplementary file 6 [file Image1.PDF]

Figure S1. Details on the twelve haplotypes associated to the averaged speed. Frequency of twelve significant haplotypes centred on SNP BIEC_11782. The haplotypes are ordered by decreasing frequency. Among the 4 most frequent haplotypes, 2 are negatively associated with the speed and 2 are posivlely associated with the speed but less frequent. The last 6 haplotypes are significant but their effect was poorly estimated. Each haplotype was define by a set of 7 SNP centred on SNP BIEC_11782 (Chr1: 25715334).

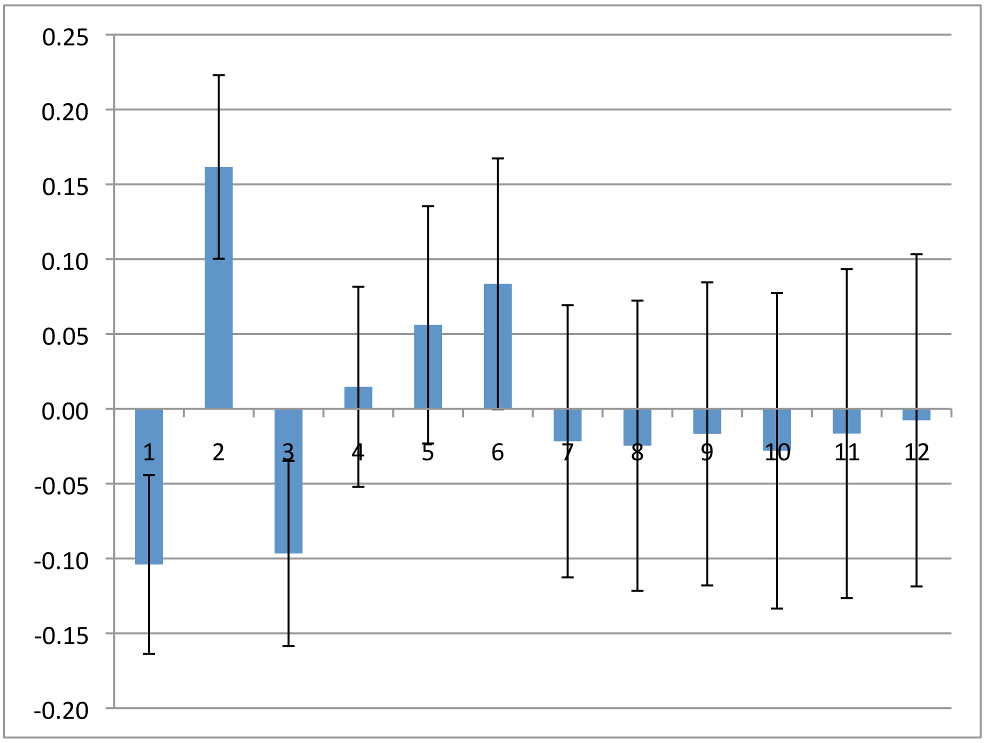

Characteristics of the 12 haplotypes

Position #on the figure	Haplotype	Effect on speed (phenotypic sd)	Standard error	Number of haplotypes
1	AACGGAA	-0.104	0.060	349
2	AACGGAG	0.162	0.061	279
3	AACGGGG	-0.097	0.062	234
4	GCCGGGG	0.015	0.067	152
5	AAAAAGG	0.056	0.079	62
6	GACGGGG	0.084	0.084	52
7	GCCGGAA	-0.022	0.091	32
8	AAAAAAA	-0.025	0.097	16
9	GACGGAG	-0.017	0.101	8
10	GCCGGAG	-0.028	0.105	7
11	GACGGAA	-0.017	0.110	2
12	AAAAAAG	-0.008	0.111	1

Linkage desequilibrium (r^2) between adjacent SNP: the ld was 1 for adjacent SNP but not for the two other distal SNP on each side.

	BIEC2_11772	BIEC2_11774	BIEC2_11778	BIEC2_11782	BIEC2_11783	BIEC2_11793	BIEC2_11799
BIEC2_11772	.	.	.	.	.	.	.
BIEC2_11774	0.71	.	.	.	.	.	.
BIEC2_11778	0.02	0.01	.	.	.	.	.
BIEC2_11782	0.02	0.01	1.00	.	.	.	.
BIEC2_11783	0.02	0.01	1.00	1.00	.	.	.
BIEC2_11793	0.21	0.14	0.03	0.03	0.03	.	.
BIEC2_11799	0.07	0.03	0.00	0.00	0.00	0.37	.

Tweleve haplotypes centred on SNP BIEC2_11782. The three central SNP are most frequently l CGG or less frequently AAA.

SNP ID	Position	Most frequent allele	Less frequent allele	MAF
BIEC2_11772	25681332	A	G	21%
BIEC2_11774	25688764	A	C	16%
BIEC2_11778	25715222	C	A	6%
BIEC2_11782	25715334	G	A	6%
BIEC2_11783	25715394	G	A	6%
BIEC2_11793	25757104	A	G	43%
BIEC2_11799	25802892	G	A	34%
