# Supplementary figures and images for: Endurance Exercise Ability in the Horse: A Trait with Complex Polygenic Determinism
Source: Front Genet. 2017 Jun 28;8:89. doi: 10.3389/fgene.2017.00089 (PMC5488500; doi:10.3389/fgene.2017.00089)

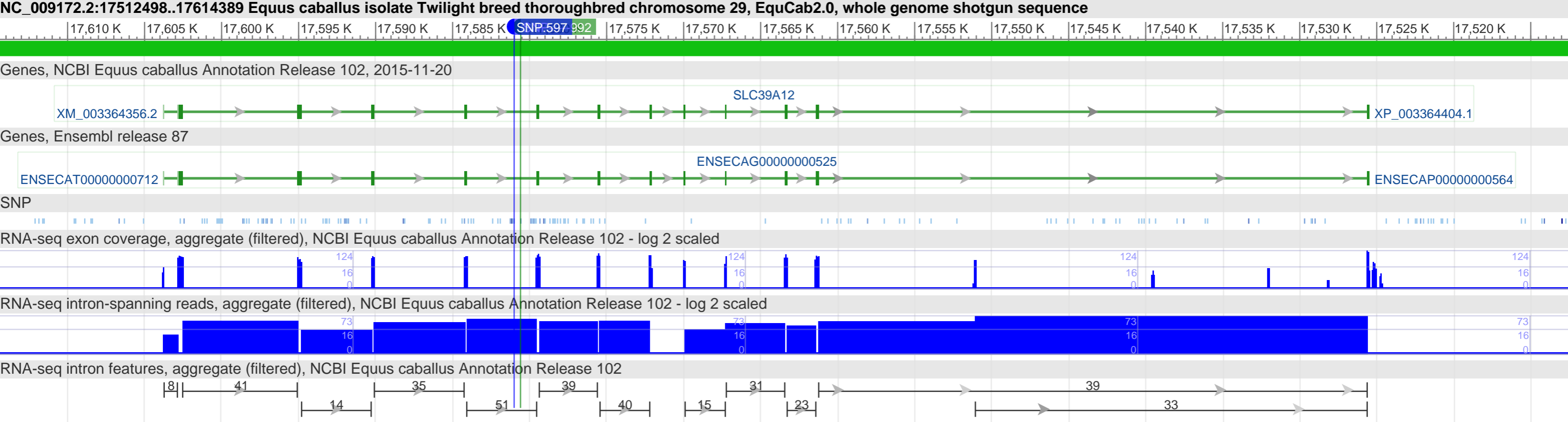

Supplement: Supplementary file 8 [file Image3.PDF]
