# Supplementary material for: Endurance Exercise Ability in the Horse: A Trait with Complex Polygenic Determinism
Source: Front Genet. 2017 Jun 28;8:89. doi: 10.3389/fgene.2017.00089 (PMC5488500; doi:10.3389/fgene.2017.00089)
Supplement: Supplementary file 7 [file Image2.PDF]

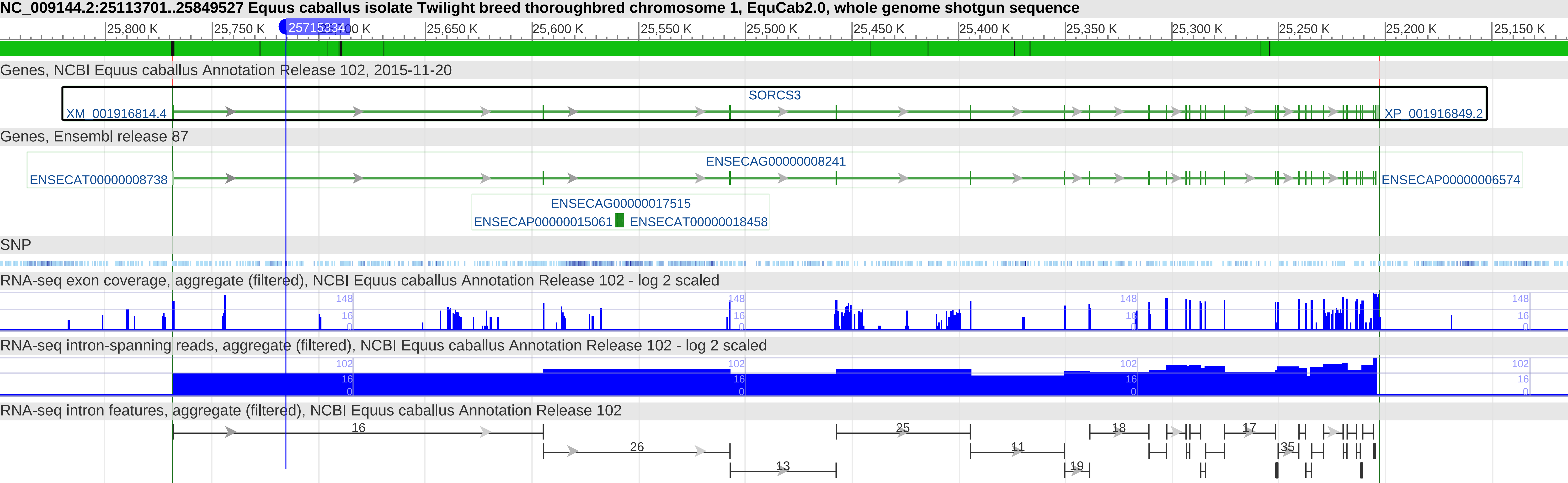
NC_009144.2:25113701..25849527 Equus caballus isolate Twilight breed thoroughbred chromosome 1, EquCab2.0, whole genome shotgun sequence
25,800 K
25,750 K
25715334
25,650 K
25,600 K
25,550 K
25,500 K
25,450 K
25,400 K
25,350 K
25,300 K
25,250 K
25,200 K
25,150 K
Genes, NCBI Equus caballus Annotation Release 102, 2015-11-20
SORCS3
XM_001916814.4
XP_001916849.2
Genes, Ensembl release 87
ENSECAG00000008241
ENSECAT00000008738
ENSECAP00000006574
ENSECAG00000017515
ENSECAP00000015061
ENSECAT00000018458
SNP
RNA-seq exon coverage, aggregate (filtered), NCBI Equus caballus Annotation Release 102 - log 2 scaled
148
16
0
RNA-seq intron-spanning reads, aggregate (filtered), NCBI Equus caballus Annotation Release 102 - log 2 scaled
102
16
0
RNA-seq intron features, aggregate (filtered), NCBI Equus caballus Annotation Release 102
16
25
18
17
26
11
35
13
19
